# Circulating CD8^+^ mucosal‐associated invariant T cells correlate with improved treatment responses and overall survival in anti‐PD‐1‐treated melanoma patients

**DOI:** 10.1002/cti2.1367

**Published:** 2022-01-10

**Authors:** Victoria M Vorwald, Dana M Davis, Robert J Van Gulick, Robert J Torphy, Jessica SW Borgers, Jared Klarquist, Kasey L Couts, Carol M Amato, Dasha T Cogswell, Mayumi Fujita, Moriah J Castleman, Timothy Davis, Catherine Lozupone, Theresa M Medina, William A Robinson, Laurent Gapin, Martin D McCarter, Richard P Tobin

**Affiliations:** ^1^ Division of Surgical Oncology Department of Surgery University of Colorado Anschutz Medical Campus Aurora CO USA; ^2^ Division of Medical Oncology Department of Medicine University of Colorado Anschutz Medical Campus Aurora CO USA; ^3^ The Netherlands Cancer Institute Amsterdam The Netherlands; ^4^ Department of Immunology and Microbiology University of Colorado Anschutz Medical Campus Aurora CO USA; ^5^ Department of Dermatology University of Colorado Anschutz Medical Campus Aurora CO USA; ^6^ Department of Biochemistry and Molecular Genetics School of Medicine University of Colorado Anschutz Medical Campus Aurora CO USA; ^7^ Division of Biomedical Informatics and Personalized Medicine Department of Medicine University of Colorado Anschutz Medical Campus Aurora CO USA

**Keywords:** immunotherapy, MAIT cells, melanoma, overall survival

## Abstract

**Objectives:**

While much of the research concerning factors associated with responses to immune checkpoint inhibitors (ICIs) has focussed on the contributions of conventional peptide‐specific T cells, the role of unconventional T cells, such as mucosal‐associated invariant T (MAIT) cells, in human melanoma remains largely unknown. MAIT cells are an abundant population of innate‐like T cells expressing a semi‐invariant T‐cell receptor restricted to the MHC class I‐like molecule, MR1, presenting vitamin B metabolites derived from bacteria. We sought to characterise MAIT cells in melanoma patients and determined their association with treatment responses and clinical outcomes.

**Methods:**

In this prospective clinical study, we analysed the frequency and functional profile of circulating and tumor‐infiltrating MAIT cells in human melanoma patients. Using flow cytometry, we compared these across metastatic sites and between ICI responders vs. non‐responders as well as healthy donors.

**Results:**

We identified tumor‐infiltrating MAIT cells in melanomas across metastatic sites and found that the number of circulating MAIT cells is reduced in melanoma patients compared to healthy donors. However, circulating MAIT cell frequencies are restored by ICI treatment in responding patients, correlating with treatment responses, in which patients with high frequencies of MAIT cells exhibited significantly improved overall survival.

**Conclusion:**

Our results suggest that MAIT cells may be a potential predictive marker of responses to immunotherapies and provide rationale for testing MAIT cell‐directed therapies in combination with current and next‐generation ICIs.

## Graphical abstract

While the number of circulating MAIT cells is reduced in melanoma patients, circulating MAIT cell frequency is restored by immune checkpoint inhibitor treatment in responding patients, correlating with treatment responses, in which patients with high frequencies of MAIT cells exhibited significantly improved overall survival.

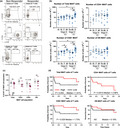

## Introduction

Melanoma characteristics, such as PD‐L1 expression, levels of immune cell infiltration, alterations in antigen processing and presentation, mutational burden and anatomic location, have been correlated with clinical responses to immune checkpoint inhibitors (ICIs).^1^ However, much remains unknown about the complexity of immune interactions that result in successful antitumor immunity, including analysis of mucosal‐associated invariant T (MAIT) cells, which is missing in most previous studies. Unlike conventional T cells that express highly variable T‐cell antigen receptors (TCRs) recognising peptide antigens presented by highly polymorphic conventional HLA class I and II molecules, MAIT cells are a population of innate‐like T cells with a restricted TCR repertoire,^2^, ^3^, ^4^ which recognises small molecules derived from microbial riboflavin synthesis presented by the non‐polymorphic MHCI‐related molecule MR1.^2^, ^4^, ^5^ MAIT cells can comprise up to 10% of circulating T cells in healthy adults and accumulate at mucosal sites including the gut, lungs and liver, where they can account for up to 50% of CD8^+^ T cells.^4^ This high frequency makes MAIT cells the most abundant currently known population of T cells with a single antigen specificity.^6^

In humans, MAIT cells are best identified by staining with MR1‐tetramers loaded with the microbial ligand 5‐(2‐oxopropylideneamino)‐6‐d‐ribitylaminouracil (5‐OP‐RU).^7^ Upon activation, MAIT cells rapidly respond by producing cytokines including tumor necrosis factor‐α (TNFα), interferon‐γ (IFNγ) and interleukin‐17 (IL)‐17, and upregulate costimulatory molecules, including CD40 ligand (CD40L, CD154).^4^, ^8^ Furthermore, MAIT cells have direct cytotoxic functions on both virally infected and tumor cells.^9^ Finally, because of their pre‐primed/memory nature, MAIT cells express high levels of several cytokine receptors, including IL‐18Rα, IL‐12R and IL‐15R, which, upon engagement, lead to MAIT cell activation in the absence of TCR triggering, resulting in rapid cytokine secretion and cytotoxic functions, including the production of perforin, granzyme B (GZMB) and granzyme K (GZMK).^4^, ^10^

To date, the role of MAIT cells in cancer remains inconclusive.^11^, ^12^, ^13^ For example, MAIT cells were found to exert tumor‐promoting functions, with improved outcomes in MAIT cell‐deficient MR1^−/−^ mice.^11^ Similarly, hepatocellular carcinoma patients with high levels of tumor‐infiltrating MAIT cells showed worse overall survival (OS) compared to those with low levels,^14^ and a regulatory subset of CD4^+^ MAIT cells was found to correlate with tumor bacterial load in human colorectal tumors.^15^ However, separate studies of oesophageal cancer, multiple myeloma and colorectal cancer patients found that higher frequencies of MAIT cells were associated with improved outcomes.^12^, ^13^, ^16^

A potential role for MAIT cells in ICI therapies is not well described. Therefore, in this prospective study, we sought to determine the frequency and functional status of MAIT cells in patients with melanoma, and whether these parameters might correlate with clinical outcomes in ICI‐treated patients. We further investigated the activation of MAIT cells in the peripheral blood and tumors from a variety of metastatic sites in melanoma patients. Our results show that MAIT cells are reduced in the circulation of melanoma patients, but that their frequency is restored in patients that respond to ICI therapy, while non‐responding patients remain low in MAIT cell count. Strikingly, high frequencies of circulating MAIT cells while on treatment are also associated with improved OS. Altogether, our results reveal circulating MAIT cell frequencies as a potential prognostic correlate of therapeutic responses to ICI immunotherapy and suggest that manipulating MAIT cells might be advantageous to anti‐melanoma immune responses.

## Results

### The frequency of MAIT cells is decreased in the circulation of melanoma patients

**Figure 1:**
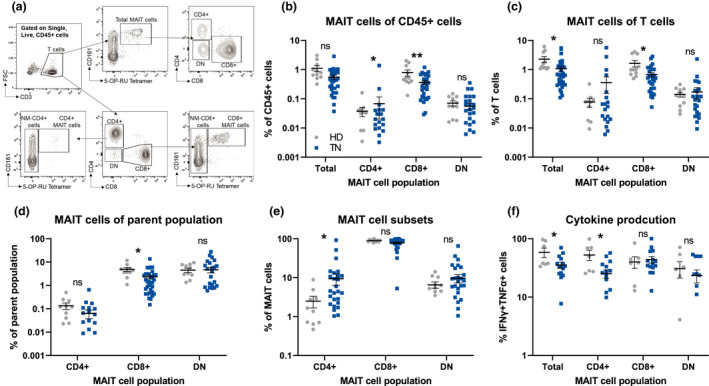
Mucosal‐associated invariant T (MAIT) cells are decreased in the circulation of melanoma patients. **(a)** Example flow cytometric gating strategy to identify MAIT cell subsets in peripheral blood. Comparisons of MAIT cell subsets as a fraction of CD45^+^ cells **(b)**, total T cells **(c)**, parent T‐cell population **(d)** and of MAIT **(e)** in healthy donors (HD, *n* = 11) and treatment naïve (TN, *n* = 33) melanoma patients. **(f)** Comparisons of the frequency of PMA/ionomycin‐stimulated polyfunctional (TNFα^+^IFNγ^+^) cells as a percentage of the described population [(HD: *n* = 11), (TN: *n* = 15)]. Double negative (DN), *P < 0.05.

We began our analysis by comparing the frequency of MAIT cell subsets in the peripheral blood of treatment naïve (TN, *n* = 33) melanoma patients, regardless of stage and healthy donors (HD, *n* = 11) with a similar age and sex distribution (Supplementary table 1). MAIT cells were identified using flow cytometry as live, CD45^+^CD3^+^CD161^+^5‐OP‐RU‐Tetramer^+^ cells, and further stratified into subsets based on CD4 and CD8 expression (Figure 1a). The frequency of circulating total and CD8^+^ MAIT cells (Figure 1b–d) was reduced in TN melanoma patients compared to HD. The reduction was apparent regardless of whether it was expressed as a proportion of total CD45^+^ (Figure 1b) cells or total CD3^+^ T cells (Figure 1c). We observed no statistically significant changes in the frequency of CD4^+^ MAIT cells (Figure 1b–d). However, the frequency of CD4^+^ MAIT cells as a proportion of MAIT cells was increased in melanoma patients than HD (Figure 1e). Conventional T cells, the majority of non‐MAIT (NM)‐CD8^+^ and NM‐CD4^+^ T cells, defined as 5‐OP‐RU‐MR1 Tetramer^−^, are usually associated with the capacity to mount antitumor immune responses.^17^ No significant differences in the frequency of either NM‐CD4^+^ or NM‐CD8^+^ T cells were observed between HD and melanoma patients (Supplementary figure 1a and b), demonstrating that the decreased frequency of MAIT cells in the blood of melanoma patients is specific to this T‐cell population.

### Melanoma‐dependent changes in MAIT cell frequencies are associated with decreased cytokine production

Human MAIT cells produce the cytokines IFNγ and TNFα as well as the effector molecules GZMB and GZMK upon activation.^4^, ^18^, ^19^ To determine whether there are functional differences in MAIT cells in melanoma patients, we measured the expression of these molecules in MAIT cells from HD and melanoma patients upon PMA/ionomycin stimulation. The percentages of polyfunctional (IFNγ^+^TNFα^+^) total MAIT cells and CD4^+^ MAIT cells were reduced in melanoma patients compared to HD, while no changes in the production of these cytokines by CD8^+^ or double‐negative (CD4^−^CD8^−^, DN) MAIT cells were observed (Figure 1f and Supplementary figure 1c). Similarly, no difference in the production of GZMB or GZMK in any circulating MAIT cell subsets between HD and melanoma patients was observed (Supplementary figure 1d–f). These data suggest that while certain MAIT cell populations from melanoma patients have decreased cytokine production, cytotoxic capacity is maintained.

### MAIT cells infiltrate melanoma tumors and express higher levels of PD‐1 within the tumor microenvironment

**Figure 2:**
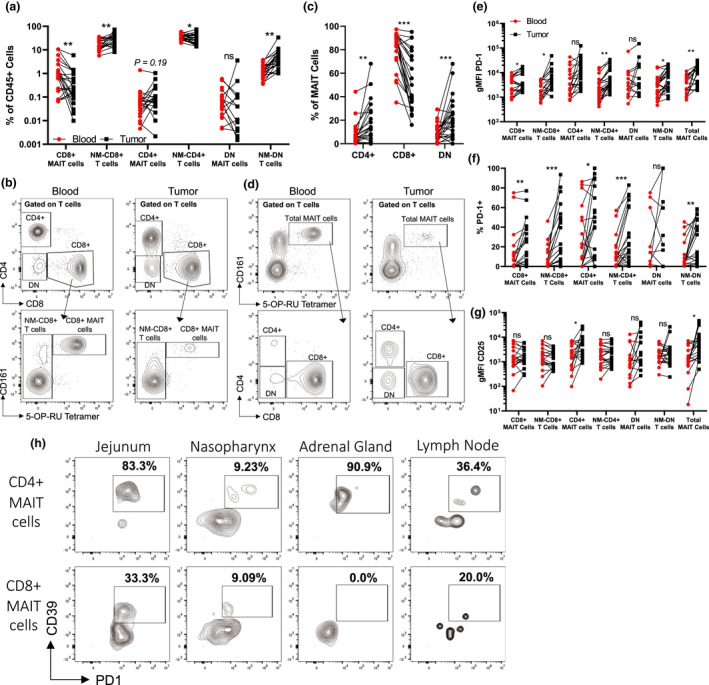
Activated mucosal‐associated invariant T (MAIT) cells infiltrate human melanoma tumors. **(a)** The frequency of MAIT cell subsets and their non‐MAIT (NM) counterparts as a percentage of the total CD45^+^ cells in the blood and infiltrating the tumors. **(a)** Example staining of MAIT cells comparing blood vs. tumor. **(c)** The ratio of each MAIT cell subset as a percentage of MAIT cells comparing blood vs. tumor. **(d)** Example staining of MAIT cells comparing blood vs. tumor. Comparisons of the geometric mean fluorescence intensity (gMFI) **(e)** of PD‐1 or **(f)** percentage of positive staining cells on MAIT cell subsets in the blood vs. tumor. **(g)** The gMFI of CD25 on MAIT cell subsets in the blood vs. tumor. **(h)** Example staining of CD39^+^PD‐1^+^ cells in different metastatic sites gated on CD4^+^ or CD8^+^ MAIT cells. Blood samples were collected from 24 patients at the time of surgery. Double negative (DN), *P < 0.05, **P < 0.01, ***P < 0.001.

We next analysed the frequency and phenotype of MAIT cells from matched tumor and blood samples in stage IV patients collected at the time of surgery. While MAIT cells could be detected within melanoma metastases, their frequencies were generally lower than the circulation, and this effect was particularly evident for CD8^+^ MAIT cells (Figure 2a and b). In contrast, a trend towards an increased ratio of CD4^+^ MAIT cells within tumors was observed (Figure 2a), with CD4^+^ and DN MAIT cells constituting a significantly greater percentage of the tumor‐infiltrating MAIT cell population than in the circulation (Figure 2c and d). Differences in chemokine receptor expression, tissue specificity, or activation requirements between CD4^+^ vs. CD8^+^ MAIT cells remain open questions and may, at least in part, explain the differences in the frequencies of MAIT cell subsets in the circulation and tumor microenvironment.^20^, ^21^, ^22^

We next compared the phenotype of MAIT cells in the blood and those infiltrating melanoma metastases. Total and CD8^+^ MAIT cells as well as NM‐CD4^+^, NM‐CD8^+^, and DN T cells, but not CD4^+^ and DN MAIT cells, showed increased fractions of PD‐1‐expressing cells and levels of PD‐1 expression within the tumor microenvironment compared to circulating cells (Figure 2e and f). Interestingly, only CD4^+^ MAIT cells demonstrated increased CD25 expression, while CD154 levels were comparable on all T‐cell populations in the circulation and the tumor microenvironment (Figure 2g and Supplementary figure 1g). Increased PD‐1 expression and low levels of CD25 and CD154 on conventional CD8^+^ T cells have been associated with chronic TCR stimulation in an immunosuppressive microenvironment.^23^, ^24^ Therefore, the phenotype of tumor‐infiltrating MAIT cells could reflect chronic TCR stimulation, although we cannot exclude that other mechanisms, such as tumor microenvironment or currently unknown factors, may also be responsible. It is important to note that melanoma tumors analysed in this study were not responding to therapy at the time they were surgically excised, and MAIT cells may be significantly different in lesions that respond to therapy.

Non‐MAIT, MR1‐restricted T cells were recently shown to recognise tumor cells in an MR1‐dependent manner,^25^ suggesting the existence of tumor‐derived antigens presented by MR1. Whether such tumor‐derived compounds also contain MAIT cell antigens is currently unknown. Another recent report demonstrated that the frequency of MAIT cells co‐expressing CD39 and PD‐1 correlates with TCR signalling driven by intratumoral bacteria.^15^ Such bacteria could potentially represent a source of MAIT antigens in the tumor. In agreement with this possibility, we observed higher frequencies of CD4^+^, and to a lesser extent CD8^+^, CD39^+^PD‐1^+^ MAIT cells in melanoma tumors from mucosal sites (nasopharynx and jejunum). However, MAIT cells with a similar phenotype were also observed in metastases to the adrenal gland and axillary lymph node, which are not normally associated with bacterial colonisation (Figure 2h). Thus, the identification of CD39^+^PD‐1^+^ MAIT cells in our samples might be indicative of the recognition of unknown tumor‐derived MAIT antigen(s). Experiments are underway to further explore this possibility. As CD39 expression has been associated with T‐cell exhaustion,^26^ these results may indicate an exhausted MAIT cell phenotype.^14^

### ICI treatment is associated with an increased frequency of circulating CD8^+^ MAIT cells in melanoma patients responding to therapy

**Figure 3:**
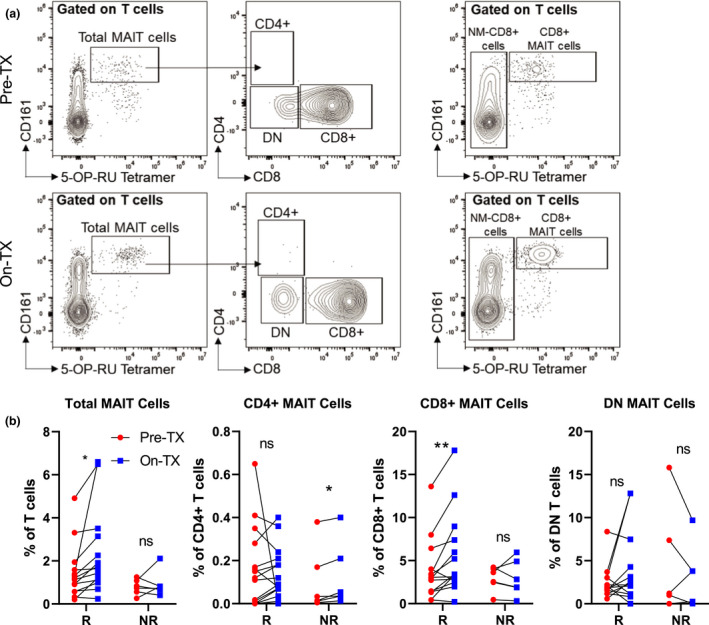
Mucosal‐associated invariant T (MAIT) cells increase in frequency in the circulation of melanoma patients during anti‐PD‐1 therapy. **(a)** Example flow cytometric data characterising the frequency of circulating MAIT cell subsets prior to treatment (Pre‐TX) and after the third anti‐PD‐1 treatment (On‐TX) in stage IV melanoma patients. **(b)** Comparisons of the labelled circulating MAIT cell subsets comparing Pre‐TX and On‐TX samples between responding (R, *n* = 14) and non‐responding (NR, *n* = 7) patients. Response to anti‐PD‐1 was characterised using RECIST1.1 criteria with responders identified as complete response (CR) or partial response (PR), while non‐responders were identified as stable disease (SD) or progressive disease (PD). Double negative (DN), *P < 0.05, **P < 0.01.

We next sought to determine whether the decreased frequency of MAIT cells observed in the circulation of melanoma patients could be influenced by ICI immunotherapy and whether this might correlate with responses to treatment. We first analysed the frequency of circulating MAIT cell subsets, comparing longitudinally collected pre‐treatment and on‐treatment [following the third anti‐PD‐1 (pembrolizumab) infusion] time points in responding (R, *n* = 14) and non‐responding (NR, *n* = 6) melanoma patients. We found that the frequency of total MAIT cells increased in the responders but not in the non‐responders (Figure 3a and b). In addition, closer examination of MAIT cell subsets revealed a statistically significant increase in the frequency of circulating CD8^+^ MAIT cells in responders but not in non‐responders, with a reciprocal increased frequency of CD4^+^ MAIT cells in non‐responders but not in responders, while there were no changes in the frequency of DN MAIT cells in either patient population (Figure 3a and b). This result corroborates studies using MAIT cells from multiple myeloma patients showing increased MAIT cell proliferation in the presence of anti‐PD‐1.^27^

**Figure 4:**
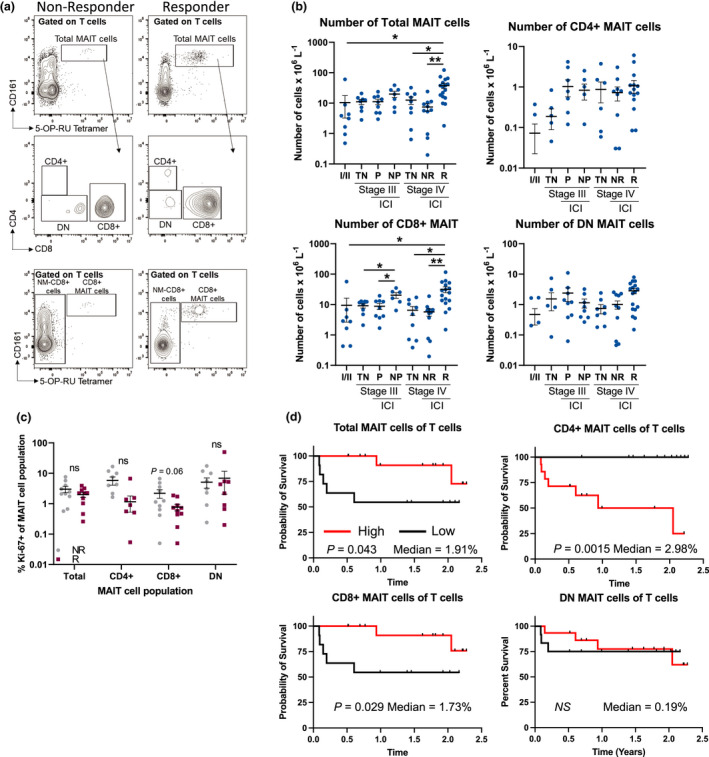
The frequency of total and CD8^+^ MAIT cells is positively associated with improved clinical responses and overall survival in melanoma patients. **(a)** Example staining of circulating MAIT cell subsets in responding vs. non‐responding melanoma patients. **(b)** Comparisons of the absolute number of total, CD4^+^, CD8^+^ and double‐negative (DN) MAIT cell populations in early stage (I/II, *n* = 16), stage III (*n* = 25) [treatment naïve (TN), progressed on treatment (P), did not progress on therapy (NP), immune checkpoint inhibitor (ICI)] or stage IV (*n* = 37) [treatment naïve (TN), non‐responding (NR), responding (R), immune checkpoint inhibitor (ICI)]. **(c)** Comparisons of the frequency of proliferating cells (Ki‐67^+^) in anti‐PD‐1 non‐responders (NR, *n* = 10) compared to responders (R, *n* = 10). **(d)** Kaplan–Meier curves comparing the survival of stage IV melanoma patients who received at least three doses of anti‐PD‐1 therapy (*n* = 27) based on the median frequency of the listed MAIT cell population. The median for each cellular population is indicated on each graph. Clinical characteristics for all patients in this analysis can be found in Supplementary tables 2–5. All treated patients received at least three doses of ICI prior to sample collection. Survival was calculated from the time of the blood draw and all blood draws occurred, while the patients were still receiving therapy. Response to ICIs was characterised using RECIST1.1 criteria with responders identified as complete response (CR) or partial response (PR), while non‐responders were identified as stable disease (SD) or progressive disease (PD).

To further determine whether the frequency of circulating MAIT cell subsets was altered in early‐stage disease (stage I/II), advanced disease (stage III and stage IV), or in patients who respond to anti‐PD‐1 therapies compared to those who do not, we analysed a larger cohort of patients with data collected at varying single time points during their treatment. We found that total MAIT cells, expressed as an absolute number or a proportion of total T cells in the circulation, are increased in stage IV patients who respond (*n* = 17) to ICI therapies compared to non‐responders (*n* = 11) and treatment naïve patients (*n* = 9; Figure 4a and b and Supplementary figure 2a). While the frequencies and absolute numbers of NM‐CD8^+^ T cells, NM‐CD4^+^ T cells and either DN or CD4^+^ MAIT cells were not significantly different between responders and non‐responders (Figure 4a and b and Supplementary figure 2a and b), we found that both the frequency and absolute numbers of CD8^+^ MAIT cells are significantly increased in responders compared to non‐responders (Figure 4a and b and Supplementary figure 2a and b). This was also apparent for stage III patients who did not progress (NP) while on therapy (*n* = 7) compared to those who progressed (P) while on therapy (*n* = 10) and TN patients (*n* = 8). Surprisingly, the increase in circulating MAIT cells in responding patients was not because of increased proliferation of the MAIT cells (Figure 4c). Although age and body mass index (BMI) have been shown to correlate with the frequency of MAIT cells in healthy individuals,^4^ such correlations were not observed in melanoma patients regardless of whether they had received an ICI or not (Supplementary figure 3a and b). These results suggest that the presence of the melanoma is directly affecting MAIT cells, altering their frequency and/or localisation. In addition, we observed no statistically significant differences in the frequencies of MAIT cell subsets when comparing stage III and IV patients who received either single‐agent anti‐PD‐1 (pembrolizumab or nivolumab) vs. combination anti‐CTLA‐4 (ipilimumab) plus anti‐PD‐1 (nivolumab; Supplementary figure 4a).

### Circulating CD8^+^ but not CD4^+^ MAIT cells are associated with improved overall survival

The improved clinical responses that we observed in patients with high frequencies of MAIT cells were intriguing. We therefore sought to determine whether they might be associated with improved OS. To reduce the confounding factors of stage and treatment, only stage IV melanoma patients who had received ≥ 3 doses of anti‐PD‐1 and were on treatment at the time of the blood draw were included in the analysis (*n* = 27). The median follow‐up time for this group of patients was 1.5 years (0.084–2.3 years). We found that patients with high levels of either total MAIT cells or CD8^+^ MAIT cells have significantly improved OS compared to those with low frequencies of these populations (Figure 4d). In contrast, patients with high frequencies of circulating CD4^+^ MAIT cells have significantly decreased OS (*P* < 0.0015; Figure 4d). Double‐negative MAIT cells were not associated with overall survival (Figure 4d). Other clinical characteristics such as primary melanoma subtype and treatment (single‐agent anti‐PD‐1 vs combination anti‐CTLA‐4 plus anti‐PD‐1) were not significantly associated with OS in this subset of patients (Supplementary figure 4b and c, Supplementary tables 2–5).

## Discussion

Our results demonstrate that the frequencies of different MAIT cell subsets in the circulation of melanoma patients undergoing ICI immunotherapy represent a potential novel prognostic correlate of immunotherapy success. We acknowledge the small number of patients as a limitation of this study. However, we believe that these results are important and open novel lines of future research. These findings corroborate and further expand upon a recent study that found similar results in melanoma patients.^28^ The role of MAIT cells in cancer remains debatable and might vary depending upon the nature of the cancer as well as the treatment administered. However, the strikingly improved OS of patients with high levels of circulating CD8^+^ MAIT cells provides strong rationale for further investigation into the potential role of MAIT cells in antitumor immunity. Previous studies have not always separated MAIT cells into subsets based on CD4 and CD8 expression, which may partially explain some discrepancies regarding the role of MAIT cells in cancer.^11^, ^14^

In this study, we found that CD8^+^ MAIT cells were positively associated with clinical responses and positively correlated with improved OS in melanoma patients. By contrast, increased frequency of CD4^+^ MAIT cells negatively correlated with OS, suggesting major differences in functions between these two subsets. These data highlight the need for a greater understanding of the functional differences between MAIT cell subsets in humans. Studies in our laboratory are ongoing to examine what such differences might be and how each subset may contribute to both response to therapy as well as OS. It will also be of interest to determine whether MAIT cells can directly kill melanoma tumor cells or whether they play an indirect role in antitumor immunity. Answers to these questions will be informative and might open new therapeutic avenues aimed at manipulating MAIT cell functions during immunotherapy treatments. Although we did not examine the microbiome in this patient cohort, it is tempting to speculate that the correlations between the contents of the microbiome and responses to anti‐PD‐1 immunotherapy might be, at least partly, mediated by microbial effects on MAIT cells.^29^ Nevertheless, this study should be interpreted considering its limitations, such as the relatively low numbers of matched samples from patients pre‐ and on‐therapy. Despite these limitations, our results highlight the potential role of MAIT cells in antitumor immunity.

## Methods

### Patients

Melanoma patients were identified at The University of Colorado Cancer Center. The clinical characteristics of all patients enrolled in this study can be found in Supplementary table 1. Peripheral blood was obtained from healthy adult donors from the Vitalant Research Institute, Denver or the University of Colorado Anschutz Medical Campus Clinical and Translational Sciences Institute COMIRB protocol #17‐2159. Peripheral blood was collected in acid citrate Vacutainer tubes (BD Biosciences, Franklin Lakes, NJ, USA). Clinical responses to ICI therapy were measured in stage IV patients according to RECIST1.1 criteria by a certified radiologist and the treating oncologist using standard of care (SOC) clinical imaging. Clinical responses in stage III patients were assessed by the treating oncologist based on the presence or absence of disease progression. Melanoma tumor samples were collected as part of SOC surgical resections. Absolute numbers of different cell types were calculated from complete blood counts (CBC) performed as part of SOC clinical visits on the same day as the research blood draws for this study.

### Patient consent for publication

All patients provided written informed consent for sample and clinical data collection as well as data publication.

### Ethics approval

Ethics approval to conduct this study was granted by the Colorado Multiple Institutional Review Board (COMIRB) Protocols #05‐0309, #16‐2367 and #17‐0110.

### Sample preparation

PBMCs were isolated by Ficoll (Cytiva Life Sciences, Marlborough, MA, USA) density centrifugation. Tumors were minced to produce pieces < 1 mm^3^ and then incubated with 5 µg mL^−1^ Liberase DL (Roche, Wilmington, MA, USA) at 37°C for 40 min and passed through 40 μm cell strainer to create a single cell suspension.

### Flow cytometric analysis

Mucosal‐associated invariant T cells were identified from whole blood by first incubating with the PE‐conjugated 5‐OP‐RU‐loaded MR1 tetramer (NIH Tetramer Core Facility, Atlanta, GA, USA) for 40 min at room temperature.^7^ Samples were then stained with the Live/Dead discrimination dye Ghost Dye 780 (Tonbo Biosciences, San Diego, CA, USA) and the fluorochrome‐conjugated antibodies CD3‐PerCP (SK7), CD4‐Alexafluor 700 (OKT4), CD8‐PE‐Cy7 (SK1), CD25‐BV605 (BC96), CD45‐BV510 (HI30) and CD161‐APC (HP‐3G10), CD154‐BV711 (24‐31), PD‐1‐BV421 (EH12.2H7), TCR Va7.2‐FITC (3C10; Biolegend, San Diego, CA, USA) and CD39‐BV711 (TU66, BD Biosciences) in FACS buffer (PBS + 2% FBS) for 20 min at room temperature. Erythrocytes were lysed using BD Biosciences Pharm Lyse Buffer (BD Biosciences) according to the manufacturer’s protocol.

For intracellular staining, PBMCs were incubated with phorbol 12‐myristate 13‐acetate (PMA, 50 ng mL^−1^ Sigma Aldrich, Burlington, MA, USA) and ionomycin (500 ng mL^−1^, Sigma Aldrich) in the presence of monensin (2 μm, Biolegend) for 4 h. Cells were washed twice in FACS buffer followed by staining with the MR1 tetramer and then cell surface staining with Live/Dead Ghost 780, CD3‐PerCP (SK7), CD4‐Alexafluor 700 (OKT4), CD8‐PE‐Cy7 (SK1), CD45‐BV510 (HI30) and CD161‐APC (HP‐3G10), as described above. Cells were fixed and permeabilised using the Intracellular Fixation and Permeabilization Buffer Set (eBioscience, Waltham, MA, USA) according to the manufacturer’s protocol. Fixed and permeabilised cells were stained for GZMB‐PE‐Cy7 (QA16A02), GZMK‐FITC (GM26E7), Ki‐67‐Alexafluor 488 (Ki‐67), IFNγ‐BV421 (4S.B3) and TNFα‐BV605 (Mab11), in permeabilisation buffer (eBioscience) for 20 min at room temperature. All samples were analysed on a Beckman Coulter (Indianapolis, IN, USA) CytoFlex S flow cytometer, and data were analysed using FlowJo Software (BD Biosciences).

### Statistical analysis

Statistical analyses were performed using one‐way ANOVAs and two‐tailed paired as well as unpaired *t*‐tests in Prism (GraphPad, San Diego, CA, USA). Error bars represent standard error of the mean (SEM). High and low levels of cell types were identified based on the median, with statistical significance of survival based on high or low levels determined using the Log‐rank test in Prism (GraphPad). To reduce selection bias, the pre‐treatment blood draws were excluded from analysis unless they were being directly compared. Levels of significance are denoted as follows: ns, not significant; *P < 0.05; **P < 0.01; ***P < 0.001; ****P < 0.0001.

## Conflict of Interest

The authors declare no potential conflict of interest.

## Author Contributions

**Victoria M Vorwald:** Data curation; Investigation; Methodology; Writing – original draft; Writing – review & editing. **Robert J Van Gulick:** Data curation; Investigation; Methodology; Writing – original draft; Writing – review & editing. **Robert J Torphy:** Data curation; Formal analysis; Validation; Visualization; Writing – original draft; Writing – review & editing. **Dana M Davis:** Data curation; Investigation; Methodology; Writing – original draft; Writing – review & editing. **Jessica SW Borgers:** Data curation; Investigation; Methodology; Writing – original draft; Writing – review & editing. **Jared Klarquist:** Formal analysis; Investigation; Methodology; Validation; Visualization; Writing – original draft; Writing – review & editing. **Kasey L Couts:** Investigation; Methodology; Validation; Writing – original draft; Writing – review & editing. **Carol M Amato:** Data curation; Investigation; Methodology; Writing – original draft; Writing – review & editing. **Dasha T Cogswell:** Data curation; Investigation; Methodology; Visualization; Writing – original draft; Writing – review & editing. **Mayumi Fujita:** Data curation; Formal analysis; Investigation; Writing – original draft; Writing – review & editing. **Moriah J Castleman:** Data curation; Resources; Writing – review & editing. **Timothy Davis:** Investigation; Methodology; Writing – original draft; Writing – review & editing. **Catherine Lozupone:** Formal analysis; Funding acquisition; Investigation; Writing – original draft; Writing – review & editing. **Theresa M Medina:** Conceptualization; Funding acquisition; Writing – original draft; Writing – review & editing. **William A Robinson:** Investigation; Resources; Writing – original draft; Writing – review & editing. **Laurent Gapin:** Conceptualization; Formal analysis; Funding acquisition; Project administration; Visualization; Writing – original draft; Writing – review & editing. **Martin D McCarter:** Conceptualization; Funding acquisition; Investigation; Project administration; Writing – original draft; Writing – review & editing. **Richard P Tobin:** Conceptualization; Data curation; Formal analysis; Funding acquisition; Project administration; Validation; Visualization; Writing – original draft; Writing – review & editing.

## Supporting information

Supplementary figure 1
Supplementary figure 2
Supplementary figure 3
Supplementary figure 4
Supplementary table 1
Supplementary table 2
Supplementary table 3
Supplementary table 4
Supplementary table 5
Supplementary table 6
