# Supplementary material for: Circulating CD8+ mucosal‐associated invariant T cells correlate with improved treatment responses and overall survival in anti‐PD‐1‐treated melanoma patients
Source: Clin Transl Immunology. 2022 Jan 10;11(1):e1367. doi: 10.1002/cti2.1367 (PMC8743567; doi:10.1002/cti2.1367)
Supplement: Supplementary file 1 — Supplementary figure 1 Supplementary figure 2 Supplementary figure 3 Supplementary figure 4 Supplementary table 1 Supplementary table 2 Supplementary table 3 Supplementary table 4 Supplementary table 5 Supplementary table 6 [file CTI2-11-e1367-s001.pdf]

Supplementary figure 1

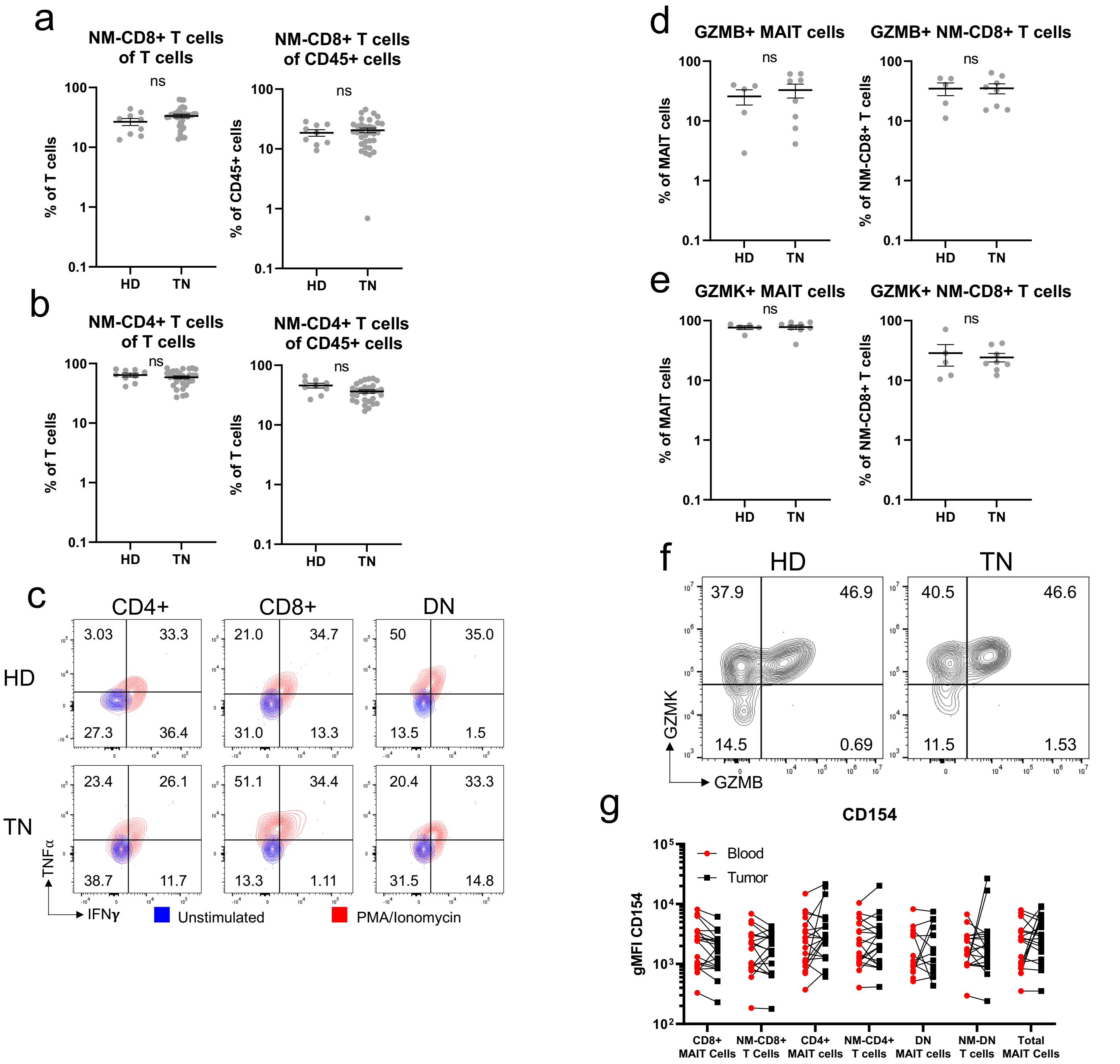

Supplementary figure 1. Comparisons of circulating non-MAIT (NM)-CD8+ (**a**) or CD4+ (**b**) T cells in healthy donors (HD, n = 11) and treatment naïve (TN, n = 33) melanoma patients. (**c**) Example TNFα vs. IFNγ staining in the listed MAIT cell population. Comparisons of the percentage of granzyme B (GZMB) (**d**) or granzyme K (GZMK) (**e**) in total MAIT cells or NM-CD8+ T cells [(HD: n = 5), (TN: n = 7)]. (**f**) Example staining of GZMB and GZMK in total MAIT cells. (**g**) Comparisons of the geometric mean fluorescence intensity (gMFI) of CD154 on circulating or tumor infiltrating MAIT cell populations (n = 24).

Supplementary figure 2

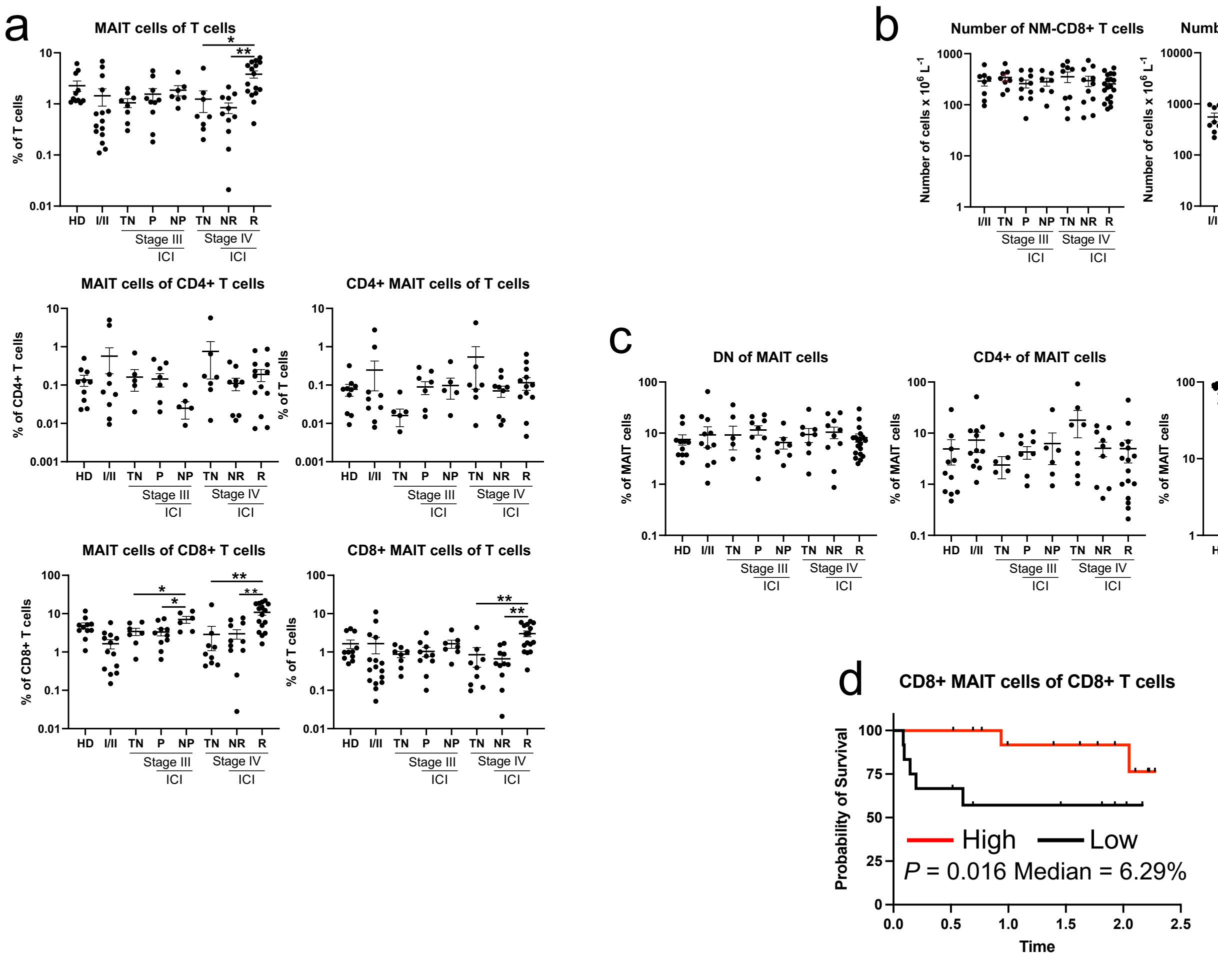

Supplementary figure 2. (**a**) Comparisons of the frequencies of MAIT cell subsets expressed as either a fraction of the labeled T cell population or the proportion of total CD3+ T cells in healthy donors (HD, n = 11), early stage (I/II, n = 16), stage III (n = 25) [Treatment Naïve (TN), progressed on treatment (P), did not progress on therapy (NP), immune checkpoint inhibitor (ICI)], or stage IV (n = 37) [Treatment Naïve (TN), non-responding (NR), responding (R), immune checkpoint inhibitor (ICI)]. (**b**) Quantification of the absolute number of circulating NM-CD8+ and NM-CD4+ T cells. (**c**) Comparisons of the ratios of DN, CD4+, and CD8+ MAIT cells as a percentage of the total MAIT cell population. (**d)** Kaplan-Meier curve comparing the survival of stage IV melanoma patients who received at least three doses of anti-PD-1 therapy (n = 27) based on the median frequency CD8+ MAIT cells as a proportion of CD8+ T cells. The median for each cellular population is indicated on each graph. Clinical characteristics for the patients in this analysis can be found in Supplementary table 6. All treated patients received at least three doses of ICI prior to sample collection. Survival was calculated from the time of the blood draw and all blood draws occurred while the patients were still receiving therapy.

Supplementary figure 3

a

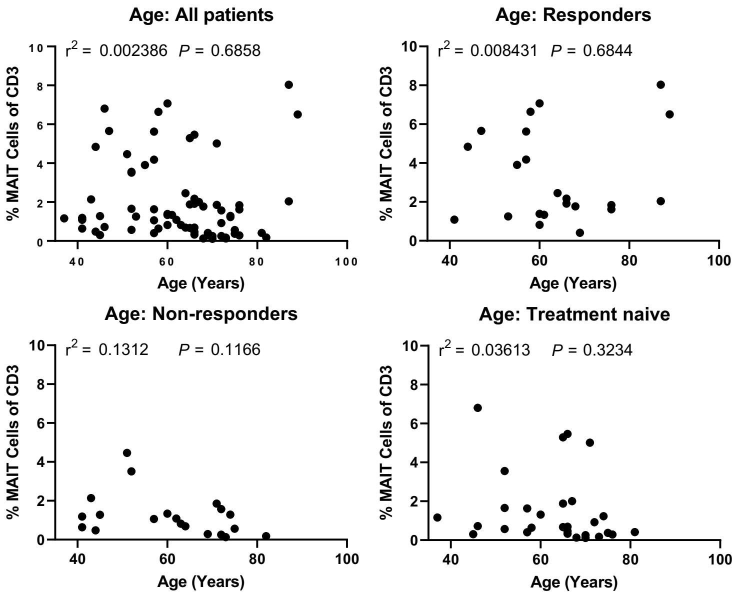

b

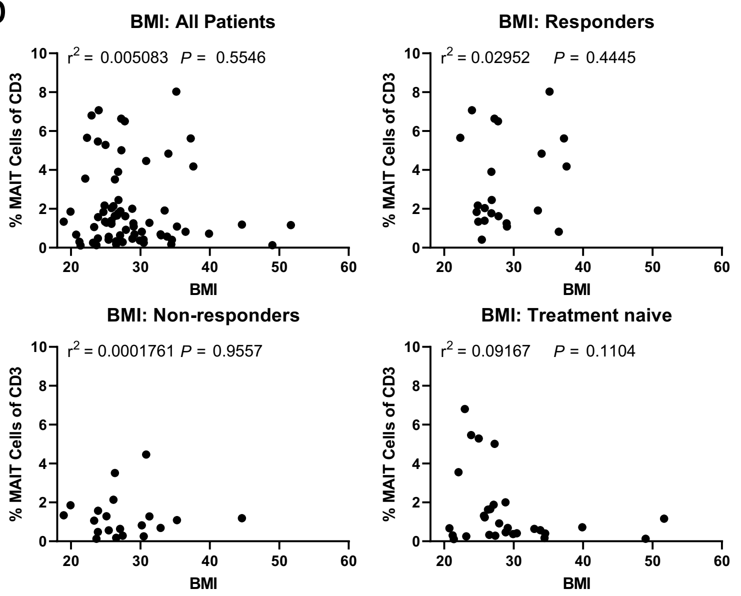

Supplementary figure 3. Correlations between the frequency of MAIT cells of total T cells (CD3+ cells) and either (**a**) age or (**b**) body mass index (BMI) in stage IV patients (n = 27). Correlations were determined using two-tailed Pearson correlation tests.

Supplementary figure 4

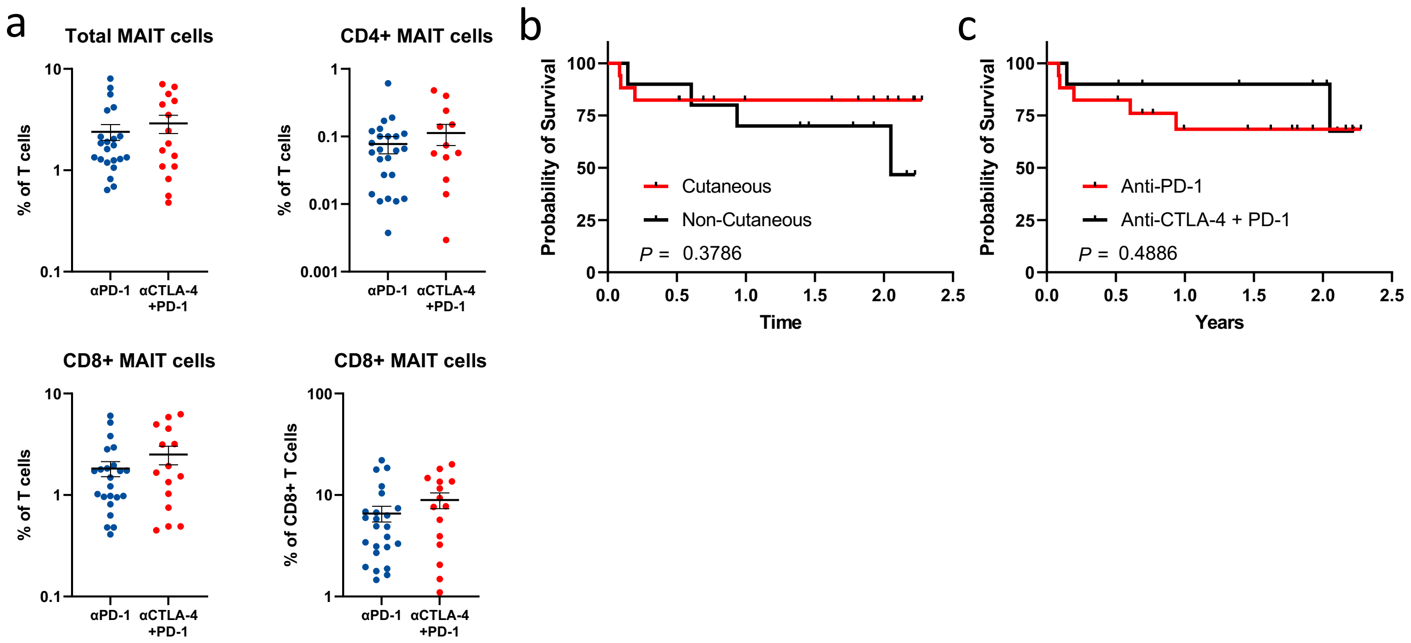

Supplementary figure 4. Treatment and disease related differences in MAIT cell frequency. (**a**) Comparisons of the frequency of MAIT cell subsets comparing single agent anti-PD-1 (αPD-1, n = 30) vs. combination anti-CTLA-4 + anti-PD-1 (αCTLA-4+PD-1, n = 15). (**b**) Kaplan-Meier curves comparing the survival of stage IV melanoma patients diagnosed with either cutaneous or non-cutaneous melanomas. (**c**) Kaplan-Meier curves comparing the survival of stage IV melanoma patients who received either single agent anti-PD-1 or combination anti-CTLA-4 + anti-PD-1.

Supplementary table 1: Patient characteristics

Patient Characteristics	HD	Stage I/II	Stage III	Stage IV
**Number of Subjects**	11	16	25	37
**Median Age at Collection (Range, Years)**	67 (51-76)	63 (46-76)	61 (37-82)	64 (41-89)
**Median BMI (Range)**		29 (21-49)	30 (20-52)	27 (19-37)
**Sex, Number (Percent)**				
Female, number (%)	4 (36)	8 (50)	11 (44)	11 (30)
Male, number (%)	7 (64)	8 (50)	14 (56)	26 (70)
**Anti-PD-1 first line Therapy**		NA	17	28
**Immune Checkpoint Inhibitor, number (%)**				
Anti-PD-1 (Pembrolizumab)		0 (0)	7 (28)	15 (41)
Anti-PD-1 (Nivolumab)		0 (0)	4 (16)	4 (11)
Combination (Ipilimumab plus Nivolumab)		0 (0)	6 (24)	9 (24)
Untreated at Sample Collection		16 (100)	8 (32)	9 (24)
**Primary Subtype, number (%)**				
Cutaneous		14 (88)	18 (72)	27 (72)
Non-cutaneous		2 (13)	7 (28)	10 (28)

[#]All patients received anti-PD-1, anti-CTLA-4, or the combination on the Q3W treatment schedule.

Supplementary table 2. Patient characteristics for Figure 4d, Total MAIT cells of T cells.

**TOTAL MAIT CELLS OF T CELLS**	**ABOVE CUTPOINT**	**BELOW CUTPOINT**
**MEDIAN AGE AT COLLECTION (RANGE, YEARS)**	59 (43-89)	69 (41-76)
**MEDIAN BMI (RANGE)**	27 (22-37)	25 (19-30)
**SEX, NUMBER (PERCENT)**		
FEMALE	3 (22)	5 (38)
MALE	11 (78)	8 (62)
**TREATMENT, NUMBER (PERCENT)**		
ANTI-PD-1 (PEMBROLIZUMAB OR NIVOLUMAB)	11 (78)	10 (77)
COMBINATION (IPILIMUMAB PLUS NIVOLUMAB)	3 (22)	3 (23)
**PRIMARY MELANOMA SUBTYPE, NUMBER (%)**		
CUTANEOUS	10 (71)	7 (54)
NON-CUTANEOUS	4 (29)	5 (38)
UNKNOWN	0 (0)	1 (8)

Supplementary table 3. Patient characteristics for Figure 4d, CD4+ MAIT cells of T cells.

**CD4+ MAIT CELLS OF T CELLS**	**ABOVE CUTPOINT**	**BELOW CUTPOINT**
**MEDIAN AGE AT COLLECTION (RANGE, YEARS)**	65 (41-89)	58 (41-75)
**MEDIAN BMI (RANGE)**	27 (19-35)	26 (22-37)
**SEX, NUMBER (PERCENT)**		
FEMALE	1 (9)	7 (54)
MALE	13 (91)	6 (46)
**TREATMENT, NUMBER (PERCENT)**		
ANTI-PD-1 (PEMBROLIZUMAB OR NIVOLUMAB)	11 (78)	10 (77)
COMBINATION (IPILIMUMAB PLUS NIVOLUMAB)	3 (22)	3 (23)
**PRIMARY MELANOMA SUBTYPE, NUMBER (%)**		
CUTANEOUS	9 (64)	8 (61)
NON-CUTANEOUS	5 (36)	4 (31)
UNKNOWN	0 (0)	1 (8)

Supplementary table 4 Patient characteristics for Figure 4d, CD8+ MAIT cells of T cells.

CD8+ MAIT CELLS OF T CELLS	ABOVE CUTPOINT	BELOW CUTPOINT
MEDIAN AGE AT COLLECTION (RANGE, YEARS)	59 (43-89)	69 (41-76)
MEDIAN BMI (RANGE)	27 (22-37)	25 (19-30)
SEX, NUMBER (PERCENT)		
FEMALE	3 (22)	5 (38)
MALE	11 (78)	8 (62)
TREATMENT, NUMBER (PERCENT)		
ANTI-PD-1 (PEMBROLIZUMAB OR NIVOLUMAB)	11 (78)	10 (77)
COMBINATION (IPILIMUMAB PLUS NIVOLUMAB)	3 (22)	3 (23)
PRIMARY MELANOMA SUBTYPE, NUMBER (%)		
CUTANEOUS	10 (71)	7 (54)
NON-CUTANEOUS	4 (29)	5 (38)
UNKNOWN	0 (0)	1 (8)

Supplementary table 5. Patient characteristics for Figure 4d, DN MAIT cells of T cells.

**CD8+ MAIT CELLS OF T CELLS**	**ABOVE CUTPOINT**	**BELOW CUTPOINT**
**MEDIAN AGE AT COLLECTION (RANGE, YEARS)**	59 (43-89)	69 (41-76)
**MEDIAN BMI (RANGE)**	27 (22-37)	26 (19-33)
**SEX, NUMBER (PERCENT)**		
**FEMALE**	4 (29)	4 (31)
**MALE**	10 (71)	9 (69)
**TREATMENT, NUMBER (PERCENT)**		
**ANTI-PD-1 (PEMBROLIZUMAB OR NIVOLUMAB)**	11 (78)	10 (77)
**COMBINATION (IPILIMUMAB PLUS NIVOLUMAB)**	3 (22)	3 (23)
**PRIMARY MELANOMA SUBTYPE, NUMBER (%)**		
**CUTANEOUS**	9 (64)	9 (69)
**NON-CUTANEOUS**	5 (36)	3 (23)
**UNKNOWN**	0 (0)	1 (8)

Supplementary table 6. Patient characteristics for Supplementary figure 2d, CD8+ MAIT cells of CD8+ T cells.

CD8+ MAIT CELLS OF CD8+ T CELLS	ABOVE CUTPOINT	BELOW CUTPOINT
**MEDIAN AGE AT COLLECTION (RANGE, YEARS)**	59 (43-89)	68 (41-87)
**MEDIAN BMI (RANGE)**	27 (22-37)	26 (19-33)
**SEX, NUMBER (PERCENT)**		
FEMALE	4 (29)	4 (31)
MALE	10 (71)	9 (69)
**TREATMENT, NUMBER (PERCENT)**		
**ANTI-PD-1 (PEMBROLIZUMAB OR NIVOLUMAB)**	10 (71)	11 (84)
**COMBINATION (IPILIMUMAB PLUS NIVOLUMAB)**	4 (29)	2 (16)
**PRIMARY MELANOMA SUBTYPE, NUMBER (%)**		
CUTANEOUS	9 (71)	8 (61)
NON-CUTANEOUS	5 (29)	4 (31)
UNKNOWN	0 (0)	1 (8)
